# Prognostic role of neutrophil–lymphocyte ratio in nasopharyngeal carcinoma: A meta-analysis

**DOI:** 10.1371/journal.pone.0181478

**Published:** 2017-07-17

**Authors:** Yukinori Takenaka, Takahiro Kitamura, Ryohei Oya, Naoki Ashida, Kotaro Shimizu, Kazuya Takemura, Yoshifumi Yamamoto, Atsuhiko Uno

**Affiliations:** Department of Otorhinolaryngology-Head and Neck Surgery, Osaka General Medical Center, Osaka, Japan; The Ohio State University, UNITED STATES

## Abstract

**Background:**

Inflammatory markers are used to predict prognosis of nasopharyngeal carcinoma (NPC). Previous reports of neutrophil-to-lymphocyte ratio (NLR) and NPC mortality are inconsistent. This study aimed to quantify the prognostic impact of NLR on NPC.

**Methods:**

The primary outcome was overall survival (OS), and the secondary outcomes were disease-specific survival (DSS), progression-free survival (PFS) and distant metastasis-free survival (DMFS). We systematically searched electronic databases, identified articles reporting an association between NLR and NPC prognosis. Hazard ratios (HRs) and 95% confidence intervals (CIs) were extracted, and pooled HRs for each outcome were estimated using random effect models.

**Results:**

Nine studies enrolling 5397 patients were included in the analyses. NLR greater than the cutoff value was associated with poor overall survival (HR 1.51, 95% CI 1.27–1.78), disease-specific survival (HR 1.44, 95% CI 1.22–1.71), progression-free survival (HR 1.53, 95% CI 1.22–1.90), and distant metastasis-free survival (HR 1.83, 95% CI 1.14–2.95).

**Conclusions:**

Elevated NLR predicts worse OS, DSS, PFS and DMFS in patients with NPC.

## Introduction

Nasopharyngeal carcinoma (NPC) is a cancer arising from the nasopharynx epithelium. NPC differs from other head and neck cancers in its characteristics, the most distinct one being its epidemiology. NPC is very rare in most part of the world with the incidence rate of less than 1 per 100,000 person-years [1]. However, the incidence rate is as high as 3.4–21.4 in the southern part of China. Southeast Asia, North Africa, the Middle East and the Arctic are the intermediate risk regions [1]. WHO classifies NPC into three types: nonkeratinizing carcinoma, keratinizing carcinoma and basaloid squamous cell carcinoma [2]. The nonkeratinizing carcinoma, previously subdivided to differentiated and undifferentiated nonkeratinizing carcinoma, is the predominant histologic type, and is invariably associated with the Epstein–Barr virus (EBV) [3]. Because of its anatomic location, surgical treatment is not usually selected, and radiotherapy (RT) is the first-line treatment for non-metastatic NPC. According to the National Comprehensive Cancer Network guideline [4], definitive RT is recommended for stage I NPC, whereas chemoradiation therapy (CRT) is recommended for stages II–IV NPC without distant metastasis. Platinum-based chemotherapy is recommended for metastatic disease. In some institutions, induction or adjuvant chemotherapy is administered in conjunction with CRT. However, the use of induction chemotherapy or adjuvant chemotherapy is controversial [5]. Because of its toxicity and, if any, small additional effect, not all patients with advanced NPC will benefit from the additional chemotherapy. Therefore, it is important to identify patients with unfavorable prognosis, who are most likely to benefit from intensified treatment. To identify groups with poor prognosis, prognostic and predictive biomarkers have been sought.

Various biomarkers, including plasma EBV DNA, D-dimer, lactate dehydrogenase, and inflammatory markers, have been found to be associated with the prognosis of NPC [6–10]. Neutrophil–lymphocyte ratio (NLR) is an inflammatory marker, validated as a prognostic marker for various types of cancer [11]. Neutrophil and lymphocyte counts in peripheral blood are routinely measured in clinical settings, and little additional effort is needed for the calculation of NLR. However, inconsistent results have been reported for the association between NLR and prognosis of NPC [12–18].

This study was conducted with an aim to resolve this inconsistency and to quantify the impact of NLR on prognosis of NPC.

## Materials and methods

### Search strategy

This study was conducted in accordance with the guidelines for Preferred Reporting Items for Systematic Reviews and Meta-Analyses [19]. We conducted a literature search on the association of NPC and NLR in electronic databases (PubMed www.ncbi.nlm.nih.gov/pubmed‎ and Scopus www.elsevier.com/online-tools/scopus) for articles published between January 1, 2000 and December 31, 2016. The search terms were “nasopharynx,” “nasopharyngeal,” “cancer,” “carcinoma,” “malignancy,” and “neutrophil.” References in the retrieved articles were manually searched for associated studies.

### Study selection

Inclusion criteria were as follows: (1) studies reporting the prognostic impact of pretreatment NLR in peripheral blood on NPC and (2) studies where a hazard ratio (HR) and 95% confidence interval (CI) or a *p*-value for overall survival (OS), disease-specific survival (DSS), progression-free survival (PFS), or distant metastasis-free survival (DMFS) were available. We excluded nonhuman studies and those in languages other than English. Two of the authors (YT and TK) evaluated the electronically searched titles independently. All potentially relevant publications were retrieved in full. Inter-reviewer agreement was assessed by Cohen’s kappa. Disagreement was resolved by consensus.

### Data extraction

The following data were extracted: name of first author; year of publication; institution and country; number of patients; disease stage; treatment modality; cutoff methods and cutoff values for NLR; and HRs, 95% CIs, and *p* values for OS, DSS, PFS, and DMFS. The HRs, 95% CIs, and *p* values were extracted preferentially from multivariate analyses, or HRs were extracted from univariate analyses. The Newcastle–Ottawa Scale (NOS) was used to assess the quality of the included study [20], and studies with a score of 6 or more were considered high-quality studies.

### Statistical analysis

The studies differed in the inclusion criteria, cutoff values and study quality. Therefore, we conducted the present meta-analyses with a random effect model using Comprehensive Meta-Analysis version 2 (Biostat, Englewood, NJ, USA). The meta-analysis was conducted initially in all included studies for OS, DSS, PFS, and DMFS. One study divided patients into three groups and showed HRs that were compared with the lowest NLR group [13]. For the present study, mean HRs were used as representatives of the study. One study showed results for two different NLR cutoff values—median value and the 80th percentile [18]. The latter result was used for data analysis for our study. Sensitivity analysis was performed by sequential omission of each individual study. Subgroup analyses for OS were conducted for disease stage and NLR cutoff value. The comparison between subgroups was tested with the Q-test for heterogeneity. Publication bias was assessed by the funnel plot and tested by Egger’s regression intercept test. Heterogeneity was assessed by Cochran Q and *I*^2^ statistics. All statistical tests were two-sided, and statistical significance was defined by a *p*-value of <0.05.

## Results

### Literature search results

**Fig 1:**
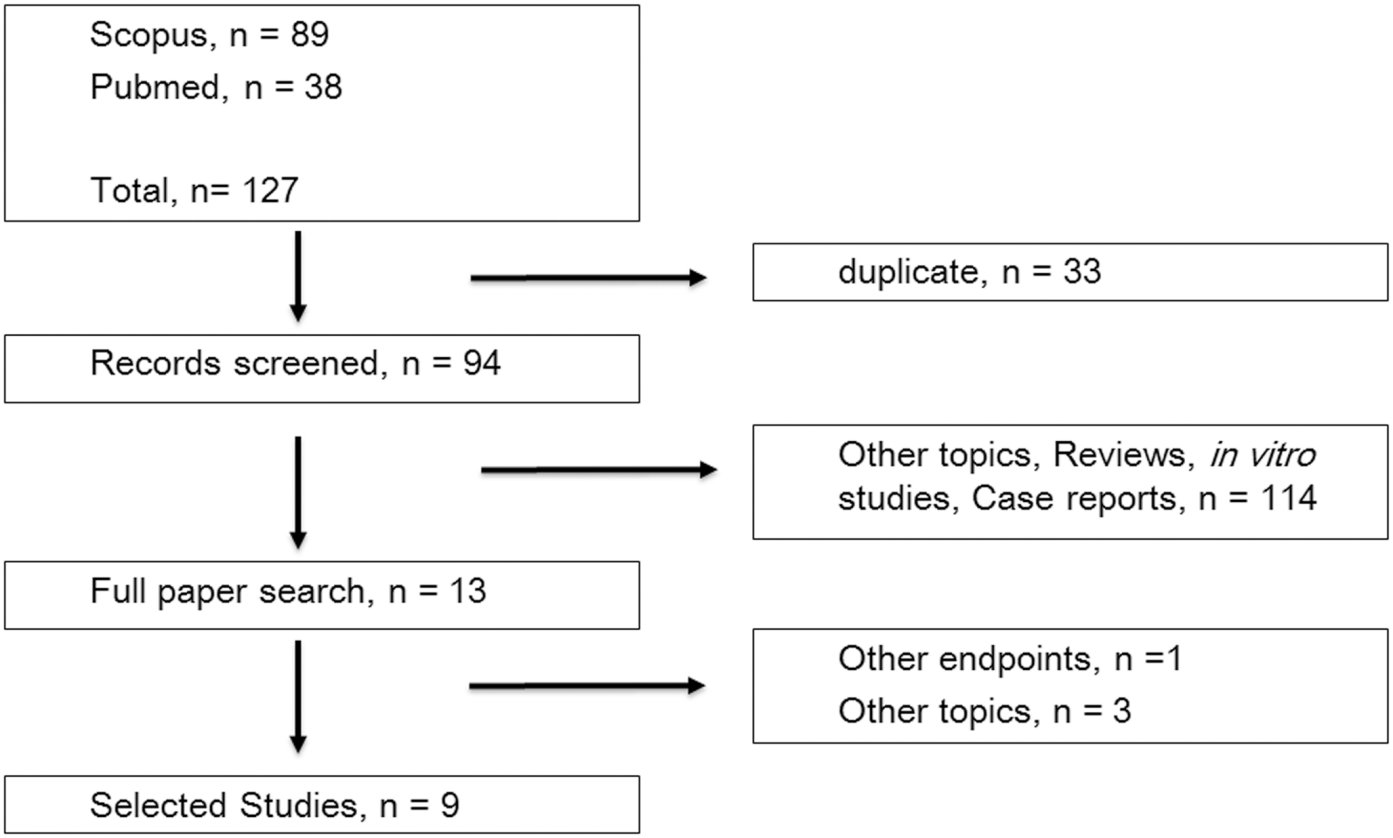
Flow diagram of article selection.

Electronic database searches retrieved 127 records (Fig 1). We examined titles and abstracts, and excluded duplicated entries, review articles, and articles written in languages other than English. The full texts of the 13 studies selected were then inspected according to exclusion criteria, and, finally, nine studies enrolling 5397 patients were included in the present study [9, 10, 12–18]. Cohen’s kappa for inter-reviewer agreement was 0.79, which indicated substantial agreement.

### Characteristics of the selected studies

Table 1 shows the characteristics of the included studies. All studies were published after 2011. The outcomes analyzed in these studies were OS in six studies [9, 13, 15–18], DSS in two studies [12, 14], PFS in three studies [13, 15, 17], and DMFS in two studies [12, 18]. The included stages were metastatic disease in two studies [15, 16], non-metastatic disease in six studies [10, 12–14, 17, 18], and mixed metastatic and non-metastatic diseases in one study [9]. The receiver operating characteristic curve was used to determine cutoff value for NLR in four studies [10, 12, 14, 17]. One report was from Singapore [18], and the rest were from China [9, 12–18]. As the result, the subjects of the present analysis belonged to the Chinese ethnicity. The mean NOS was 6 (standard deviation 1). Neutrophils and lymphocytes were counted using an automated hematology analyzer in one study [9], and the other studies [10, 12–18] did not specify the methods.

**Table 1:** Characteristics of the included studies.

year of publication	1st author	country	outcome	stage	treatment	No. of patients	cutoff method	cutoff value	analysis of HR	NOS
2011	An X	China	DSS, DMFS	non-metastatic	(C)RT	363	ROC	3.73	multivariate	6
2012	He JR	China	OS, PFS	non-metastatic	(C)RT	1410	quartile	1.54, 1.99, 2.74	multivariate (OS), univariate (PFS)	5
2013	Chang H	China	DSS	non-metastatic	(C)RT	1895	ROC	2.5	multivariate	7
2014	Chen C	China	OS, PFS	metastatic	chemotherapy	211	not reported	5	multivariate	5
2015	Jin Y	China	OS	metastatic	chemotherapy	229	median	3.60	multivariate	6
2015	Sun W	China	OS, PFS	non-metastatic	(C)RT	251	ROC	2.6 (OS), 2.7 (PFS)	multivariate	6
2016	Chua ML	Singapore	OS, DMFS	non-metastatic, locoregionally advanced	(C)RT	380	80th percentile	4.2	multivariate	6
2016	Li JP	China	OS	mixed	(C)RT	409	median	2.48	multivariate	8
2016	Li XH	China	DSS	non-metastatic	(C)RT	249	ROC	2.5	multivariate	5

Abbreviations: No, number, HR, hazard ratio, (C)RT, (chemo) radiation therapy, DSS, disease-specific survival, DMFS, distant metastasis-free survival, NOS, Newcastle–Ottawa Scale, OS, overall survival, PFS, progression-free survival, ROC, receiver operating characteristic.

### Overall survival

**Fig 2:**
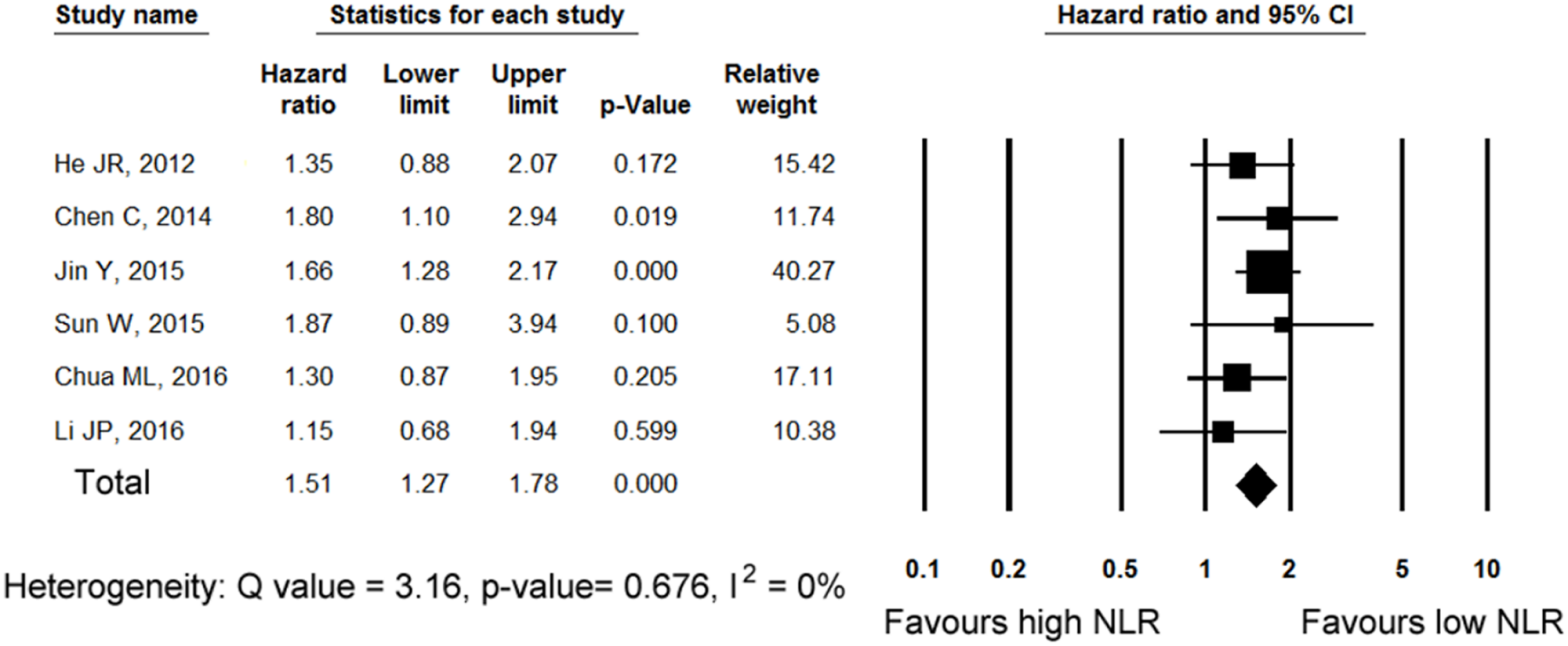
Forest plot showing hazard ratios for overall survival for neutrophil–lymphocyte ratio (NLR). The squares represent HRs for each study. The sizes of the squares and the horizontal lines crossing the squares represent the weight of the study in the meta-analysis and 95% confidence intervals, respectively. The middle and width of the diamond indicates the pooled hazard ratio and its 95% confidence interval.

Cutoff values of NLR for dichotomization ranged from 2.48 to 5 (median 3.6). Among six studies [9, 13, 15–18] reporting HRs for OS, four [9, 13, 17, 18] reported statistically non-significant HRs (*p* = 0.100 to 0.599). The pooled analysis for the six studies enrolling 2890 patients is shown in Fig 2. Overall, a higher NLR was associated with worse OS (HR 1.51, 95% CI 1.27–1.78). Significant overall HR of 1.51 was partly on account of the two significant study-specific effects of Jin et al [16] and Chen et al [15]. Result of sensitivity analysis is shown in S1A Fig. The pooled HR was significant when any single study was removed. The effect of NLR on OS between subgroups is shown in Table 2. The metastatic subgroup showed a slightly higher HR than the non-metastatic subgroup (HR 1.69 and 1.39, 95% CI, 1.34–2.14 and 95% CI, 1.05–1.82, respectively). However, the *p*-value for heterogeneity between the subgroups was not significant (*p* = 0.276). When the studies were divided into groups according to the median NLR cutoff value of 3.6, the high-cutoff subgroup had a slightly higher HR than the low-cutoff subgroup (HR 1.59 and 1.36, 95% CI, 1.30–1.94 and 95% CI, 0.86–2.14, respectively), and the impact of elevated NLR in the low-cutoff subgroup was not significant (*p* = 0.184). Moreover, the difference between subgroups was not significant (*p* for heterogeneity = 0.504).

**Table 2:** Effect of NLR on OS between subgroups.

Subgroup	No. of studies	No. of patients	HR	(95% CI)	*p*-value for HR	Q-value	*p*-value (heterogeneity)	I^2^ (%)
Stage						1.188	0.276	
non-metastatic	3	2041	1.386	(1.054–1.822)	0.020	0.733	0.693	0
metastatic	2	440	1.692	(1.341–2.136)	<0.001	0.079	0.779	0
Cut-off value						0.447	0.504	
< 3.6	2	660	1.359	(0.864–2.138)	0.184	1.098	0.295	8.906
≥3.6	3	820	1.585	(1.295–1.940)	<0.001	1.297	0.523	0

Abbreviations: No, number, HR, hazard ratio, CI, confidence interval

### Disease-specific survival, progression-free survival, and distant metastasis-free survival

**Fig 3:**
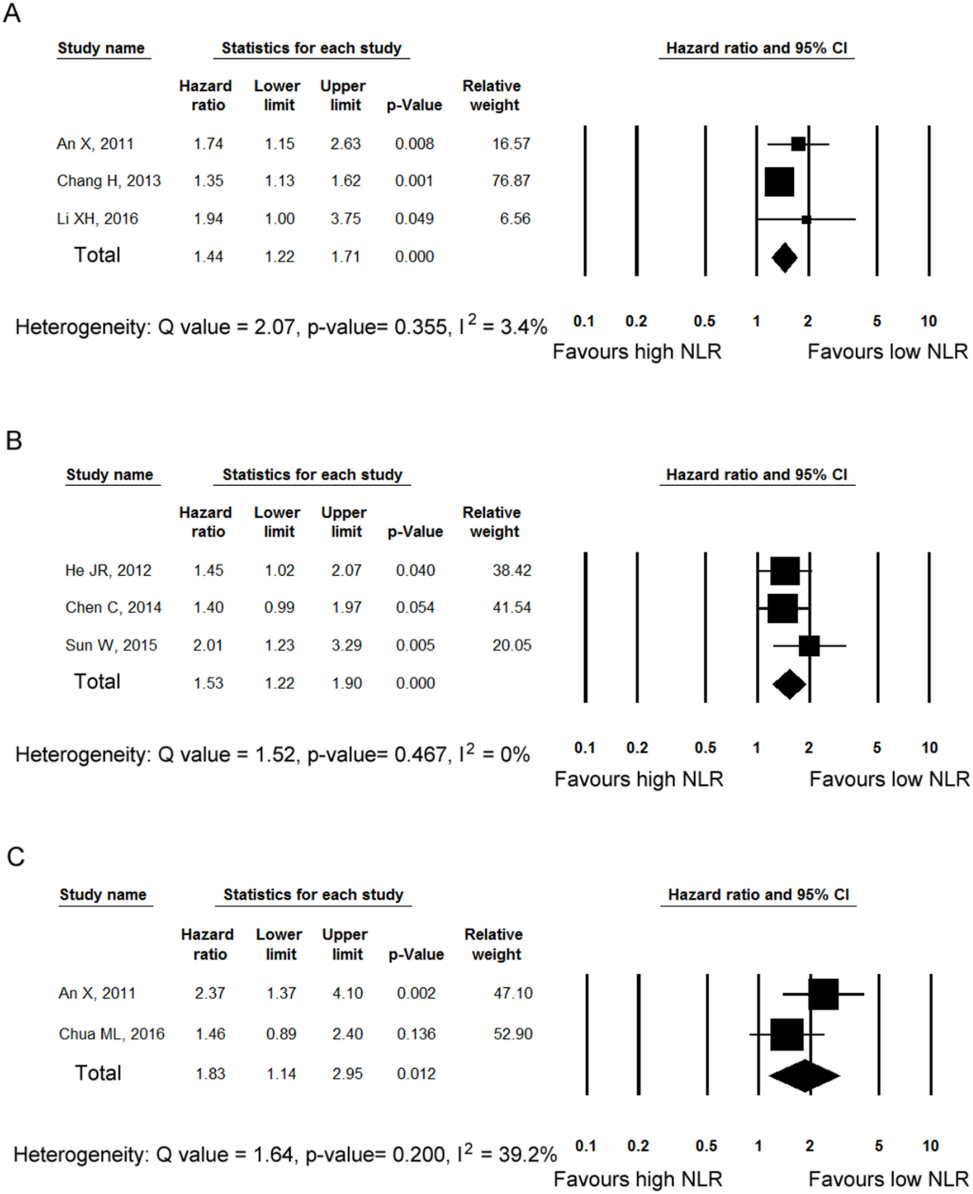
Forest plot showing hazard ratios (HRs) for disease-specific survival (A), progression-free survival (B), and distant metastasis-free survival (C) for neutrophil–lymphocyte ratio (NLR). The squares represent HRs for each study. The sizes of the squares and the horizontal lines crossing the squares represent the weight of the study in the meta-analysis and the 95% confidence intervals, respectively. The middle and width of the diamond indicates the pooled hazard ratio and its 95% confidence interval.

Fig 3A–3C show forest plots for DSS, PFS, and DMFS, respectively. Three studies [10, 12, 14] enrolling 2507 patients reported HRs for DSS. All three studies reported statistically significant HRs, and the pooled HR for DSS was 1.44 (95% CI 1.22–1.71). The results of the sensitivity analysis are shown in S1B Fig. Although the study by Chang et al. [14] contributed largely to the pooled HR, the pooled HR was still significant when the study was removed. Three studies [13, 15, 17] enrolling 1872 patients reported HRs for PFS. One [15] of the three studies reported statistically non-significant HRs, and the pooled HR for PFS was 1.53 (95% CI 1.22–1.90). Result of sensitivity analysis is shown in S1C Fig. The pooled HR was significant when any single study was omitted. The combined HR of two studies [12, 18] enrolling 743 patients for DMFS was 1.83 (95% CI 1.14–2.95).

### Publication bias

**Fig 4:**
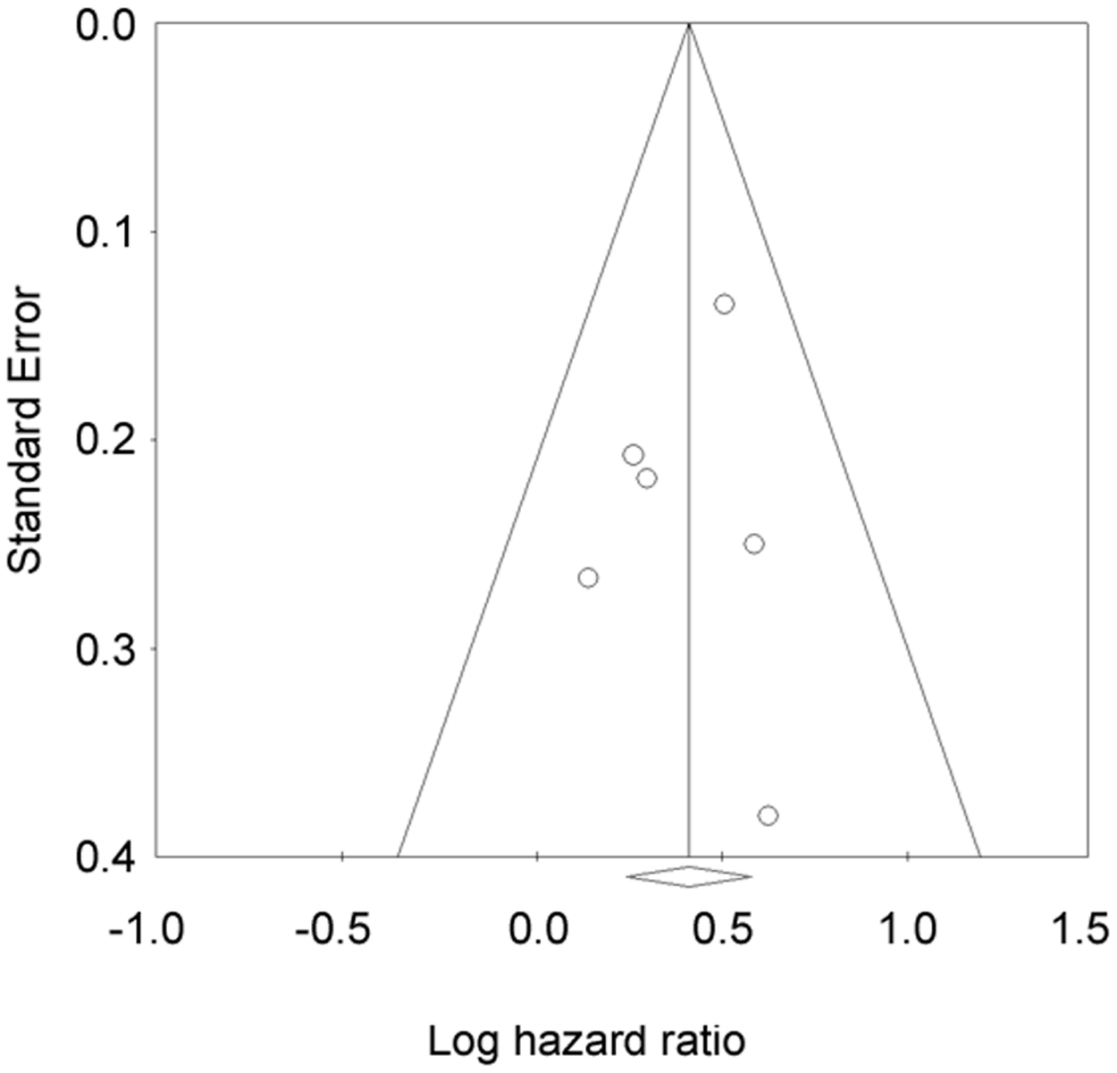
Funnel plot of hazard ratios (HRs) for overall survival for neutrophil–lymphocyte ratio.

Fig 4 shows a funnel plot of HRs for OS. The plot showed no apparent asymmetry, and Egger’s test of the intercept did not suggest publication bias (*p* = 0.715). Funnel plots for DSS, PFS, and DMFS are not shown because the numbers of studies for each outcome were small.

## Discussion

NPC has a multifactorial causation. Risk factors for NPC include EBV infection, intake of salt-preserved fish, smoking, and chronic sinonasal tract inflammation, several types of human leukocyte antigen, and other genetic variations [1, 21]. Of these factors, EBV infection contributes the most to the carcinogenesis of NPC. The EBV genome is almost universally present in tumor cells of nonkeratinizing NPC [22]. The latent EBV genes encode proteins characteristic of NPC, such as Epstein–Barr nuclear antigen 1, latent membrane proteins (LMPs) 1, 2A, and 2B [22]. Some of these EBV gene products interfere with antigen-processing, recognition, and cytotoxicity in the immunity system [23–25], resulting in immune evasion. In contrast, EBV-encoded small RNAs trigger inflammation and attract tumor-associated macrophages and T lymphocytes, leading to tumor progression [26, 27]. Thus, EBV-infected NPC cells harness immune cells and facilitate a protumorigenic inflammatory tumor microenvironment.

Because of their anti- or protumor effect, immune cells in the tumor microenvironment can be used as prognostic markers. Intratumoral CD8 T cells expressing programmed death-1 were associated with poor prognosis for NPC [28]. In contrast, tumor-associated macrophages and Foxp3+ tumor-infiltrating lymphocytes correlated with favorable prognosis for NPC [29, 30]. Quantification of immune cells in tumor tissues requires advanced techniques and considerable effort. Tissue biomarker measurement using biopsy samples is associated with a concern of heterogeneity within a tumor. Therefore, research has been undertaken to investigate the prognostic ability of circulating immune cells. NPC patients with a higher CD4 to CD8 ratio in circulating lymphocytes showed better DMFS than counterparts with a lower ratio [31]. Among patients with EBV-associated NPC, low circulating CD19+ B cells predicted poorer PFS and OS [32]. High circulating CD44+ lymphocytes was an unfavorable marker in patients with NPC treated with CRT [33]. Although the specific subtypes of immune cells in circulation are associated with tumor progression, meticulous classification of subtypes may not be required for prognostic prediction. A lower lymphocyte count in circulating blood of patients with NPC was associated with worse OS and PFS [13, 16, 17, 34, 35]. In contrast, elevated neutrophil count in those patients was associated with worse OS, DSS, and DMFS [13, 36, 37]. To simultaneously consider the positive prognostic impact of lymphocytes and negative impact of neutrophils, NLR was employed, and its association with prognosis of NPC has been investigated [9, 10, 12–18]. However, there existed some inconsistency among their results, and the prognostic ability of NLR for NPC is not conclusively determined yet.

In the present study, we aimed to assess the prognostic impact of NLR on NPC by conducting a meta-analysis of nine studies that enrolled 5397 patients. Although half of the studies regarding OS reported non-significant results, the combined HR for OS showed significantly poorer OS associated with elevated NLR. Similar results were obtained in the DSS, PFS, and DMFS analyses.

A number of meta-analyses on the association between NLR and cancer prognosis have been conducted [11, 38–48], all of which showed significant associations between elevated NLR and poor prognosis; however, the pooled HRs for OS differed considerably according to tumor sites. Pooled HRs for OS were less than 2 in cancers of the cervix. lung, prostate, kidney, esophagus, and kidney [38, 40, 42, 44, 46, 48] and were 2.03–2.56 in breast, colorectal, and liver cancers [39, 41, 47]. The pooled HR in patients with rectal cancer showed a singularly high value of 13.41 [43]. The pooled HR of 1.51 for OS in our study was comparable to those reported in many previous studies. The difference of effect size among tumor sites possibly emerges from the differences in biological characteristics of tumor cells and carcinogenic background.

The effect of elevated NLR on prognosis of NPC in this study was relatively small. To discern the group with the worse prognosis, biomarkers with more discrimination ability were desirable. Among blood-derived biomarkers, plasma EBV DNA is a reliable prognostic marker specific for NPC [6], and is also used for the detection and monitoring of NPC [49, 50]. A recent meta-analysis on the prognostic ability of pretreatment EBV DNA showed an HR of 2.78 for OS [51]. In the same report, posttreatment EBV DNA showed a further large impact (HR for OS = 5.43). Therefore, decision making based on plasma EBV DNA may be incorporated into clinical practice in the near future. However, measurement of plasma EBV DNA has not become a universally accepted clinical routine yet. Further, it is doubtful whether EBV DNA measurement will be available in all parts of the world. Therefore, other blood-derived markers readily measurable in any hospitals could be used as the second-best options. One of these candidates is serum lactate dehydrogenase (LDH). The HR of high LDH for OS in a meta-analysis was 1.79 [8], which is slightly larger than the HR of NLR in this study. Other hematologic markers, such as platelet–lymphocyte-ratio (PLR) [9, 15, 17] and lymphocyte–monocyte ratio (LMR) [10] are other candidates. Two of the studies included in our meta-analysis used PLR and NLR as covariates in the multivariate models of each study [9, 17]. In both studies, HRs of PLR for mortality were higher than those of NLR. Similarly, one study used LMR and NLR as covariates in a multivariate model, and LMR was a better prognostic indicator than NLR [10].

There are several limitations in this study. First, only nine studies were included in our analysis, and the number of studies for each outcome was further diminished. Second, some of the studies included other inflammatory markers, together with NLR, as covariates in multivariate models [9, 10, 15, 17]. These might lead to an underestimation of NLR. Third, we used summarized data of individual studies, and individual patient data were not collected. Fourth, the cutoff values for NLR were inconsistent among individual studies. This can cause heterogeneity. Because of the limited number of studies, we did not conduct a meta-regression analysis to investigate the relationship between cutoff values and the effect of NLR. Hence, we could not determine the optimal cutoff value for NLR to be used in clinical settings. In the meta-analysis for the association of NLR and the prognosis of solid tumors [11], the cutoff value of 5 showed the highest HR among various cutoff values examined. Similarly, the cutoff value of 5 employed in the study by Chen et al. [15] showed relatively high HR of 1.80 (Fig 2). Therefore, it could be better to use this cutoff value until further evidence accumulates.

In conclusion, although NLR was a significant prognostic factor for NPC, the effects of elevated NLR on OS, DSS, PFS, and DMFS were small. Therefore, further research on prognostic markers is required toward the improvement of NPC treatment.

## Supporting information

S1 Fig Sensitivity analysis of the relationship between neutrophil-lymphocyte count and overall survival, disease-specific survival and progression-free survival (A, B and C). (TIF)

S1 Table Prisma 2009 checklist. (DOC)
