# Supplementary figures and images for: Prognostic role of neutrophil–lymphocyte ratio in nasopharyngeal carcinoma: A meta-analysis
Source: PLoS One. 2017 Jul 17;12(7):e0181478. doi: 10.1371/journal.pone.0181478 (PMC5513538; doi:10.1371/journal.pone.0181478)

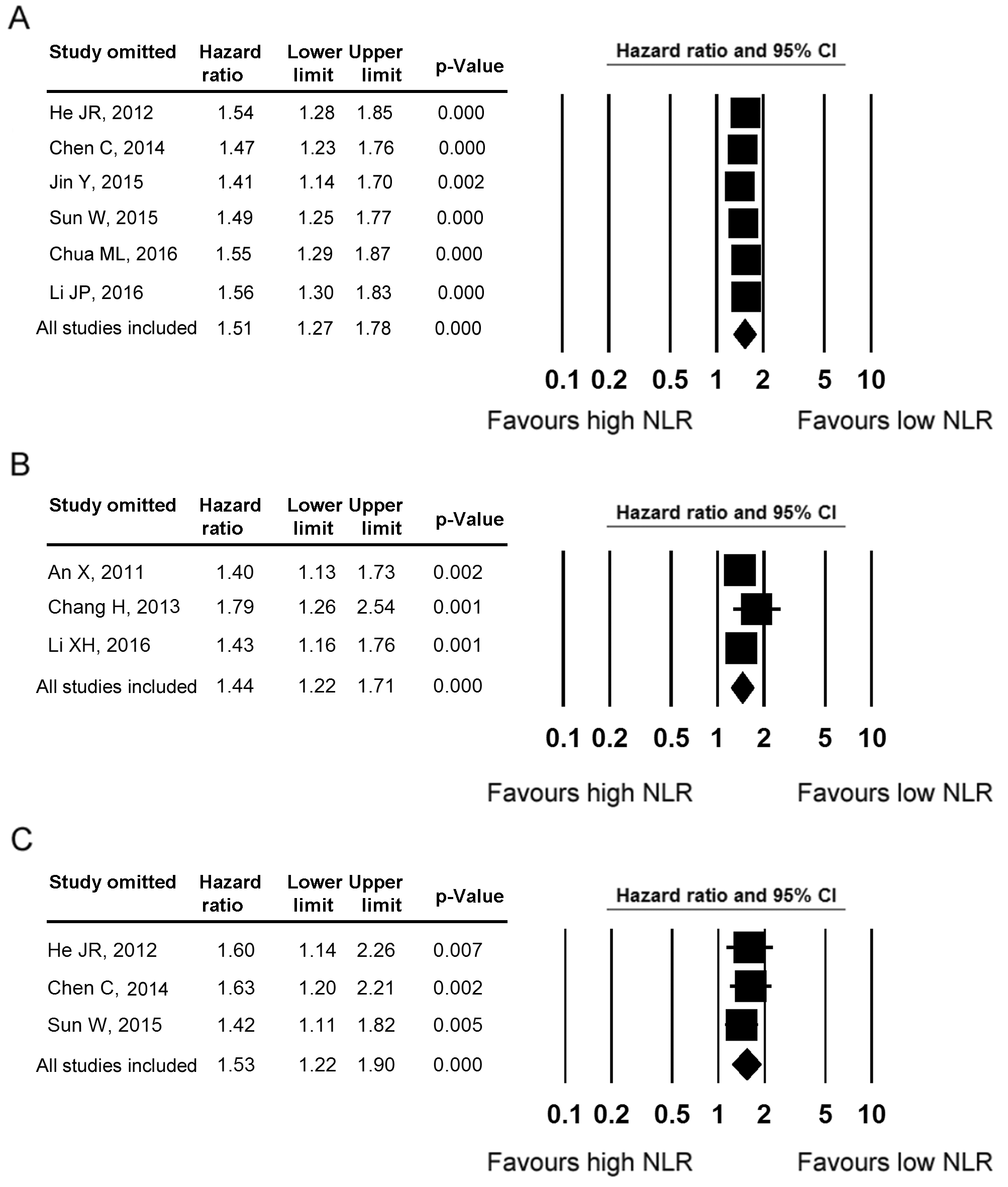

Supplement: S1 Fig — (TIF) [file pone.0181478.s001.tif]
